# Effect of second molar eruption on efficiency of maxillary first molar distalization using Carriere distalizer appliance

**DOI:** 10.1590/2177-6709.26.4.e2119146.oar

**Published:** 2021-08-27

**Authors:** Ahmed Shawky HASHEM

**Affiliations:** 1 Minia University, Faculty of Dentistry, Department of Orthodontics (Minya, Egypt).

**Keywords:** Maxillary molar distalization, Carriere distalizer, Second molar eruption

## Abstract

**Introduction:**

Maxillary molar distalization is a common approach for correcting dental Class II malocclusions.

**Objective:**

This study aimed at comparing the outcomes of maxillary first molar distalization using the Carriere appliance before and after second molar eruption.

**Methods:**

Two groups of patients with dental Class II malocclusions were treated with Carriere distalizer appliance with heavy rectangular mandibular wire and lingual arch for anchorage. Patients of the first group presented unerupted maxillary second molars during the distalization period. In the second group, maxillary second molars were in occlusion on treatment onset. Cone beam computed tomography images were taken at the beginning of treatment and after finishing molar distalization, to compare both groups regarding first molar distalization, intrusion, mesiodistal tipping, buccolingual torquing and rotation, anchorage loss and skeletal changes. Also, the treatment durations were compared.

**Results:**

The mean first molar distalization period in the first group (19.2 ± 1.6 weeks) was significantly smaller than the second group (23.3 ± 2.3 weeks). The amount of maxillary first molar distalization was significantly greater, while the amount of rotation was significantly smaller in the first group. No statistically significant differences in the amounts of maxillary first molar intrusion, mesiodistal tipping and buccolingual torquing between both groups was found. Mandibular incisor labiolingual torquing and mandibular first molar mesialization and mesiodistal tipping were significantly greater in the second group.

**Conclusions:**

Maxillary first molar distalization before maxillary second molar eruption is more efficient, with less anchorage loss than after second molar eruption.

## RESUMO

**Introdução:**

A distalização do molar superior é uma abordagem comum para a correção da má oclusão de Classe II dentária.

**Objetivo:**

O presente estudo teve como objetivo comparar os resultados da distalização do primeiro molar superior usando o aparelho Carriere antes ou após a erupção do segundo molar.

**Métodos:**

Dois grupos de pacientes com má oclusão de Classe II dentária foram tratados com o distalizador Carriere, com arco inferior retangular espesso e um arco lingual para ancoragem. Os pacientes do primeiro grupo não apresentavam os segundos molares irrompidos durante o período de distalização. No segundo grupo, os segundos molares superiores estavam em oclusão no início do tratamento. Imagens de tomografia computadorizada de feixe cônico foram obtidas no começo do tratamento e após o término da distalização do molar, para comparar ambos os grupos quanto à distalização, intrusão, inclinação mesiodistal, torque vestibulolingual e rotação do primeiro molar, além da perda de ancoragem e mudanças esqueléticas. O tempo de tratamento de ambos os grupos também foi comparado.

**Resultados:**

O tempo médio de distalização do primeiro molar no primeiro grupo (19,2 ± 1,6 semanas) foi significativamente menor que no segundo grupo (23,3 ± 2,3 semanas). A quantidade de distalização do primeiro molar superior foi significativamente maior e a quantidade de rotação foi significativamente menor no primeiro grupo. Não foram encontradas diferenças significativas entre ambos os grupos quanto à quantidade de intrusão, inclinação mesiodistal e torque vestibulolingual do primeiro molar. O torque vestibulolingual nos incisivos inferiores e a mesialização e inclinação mesiodistal do primeiro molar inferior foram significativamente maiores no segundo grupo.

**Conclusões:**

A distalização do primeiro molar superior feita previamente à erupção do segundo molar superior é mais eficiente, apresentando menor perda de ancoragem do que quando realizada após a erupção do segundo molar.

## INTRODUCTION

Dental Class II molar relationship is a frequent malocclusion that can be successfully resolved by means of extractions in at least one arch,^1^ ^,^ ^2^ using intermaxillary elastics^2^ ^,^ ^3^ or maxillary molar distalization.^4^ ^-^ ^6^ Molar distalization has become more prevalent because Class I molar relationship is achieved, a certain amount of space is gained, and tooth extractions can be prevented.^6^

Different types of appliances can be used to distalize maxillary molars including pendulum,^4^ distal jet,^5^ headgear^7^ and miniscrews.^8^ The Carriere distalizer (Henry Schein Inc., New York, NY) is a simple fixed appliance used for nonextraction Class II correction, by moving the Class II buccal segment as a block unit into a Class I occlusion.^9^ It was designed to use anchorage from the mandibular arch to create Class I molar and canine relationships.^9^ ^,^ ^10^

The distalization phase with the Carriere distalizer appliance commonly precedes full Edgewise appliances bonding, thus increasing adolescent patient’s comfort and general experience.^11^ ^,^ ^12^ The following fixed appliance therapy may be combined with orthodontic or orthopedic maxillary expansion, to refine and detail the occlusion.^12^

The type of first molar movement and the treatment timing (before or after second molar eruption) are two factors affecting not only the success, but also the efficiency of molar distalization.^13^

An unerupted maxillary second molar can perform as a fulcrum, resulting in much more evident first molar tipping than when both molars are distalized together. Accordingly, the eruption level of the second molar can have an essential influence on the first molar distalization.^14^

On the contrary, distalization of maxillary first molar alone can result in greater amount of distalization, higher movement rate and less anchorage loss than when both first and second molars are distalized concurrently.^15^ The treatment duration for maxillary first molar distalization increases if the second molar is erupted.^16,17^ Accordingly, the ideal age for maxillary first molar distalization is supposed to be prior to second molar eruption.^15^ ^-^ ^17^

Other studies concluded that the change of the first molar position and the amount of anchorage preservation are not changed significantly whether the second molar is erupted or not.^4^ ^,^ ^18^ ^,^ ^19^ The belief that the unerupted second molar represents a fulcrum, increasing the distal tipping of the distalized first molar, is unsupported.^13^

Most of the previous studies explaining the effect of maxillary second molar eruption on maxillary first molar distalization used distalizers depending on the upper arch for anchorage, and relied on two-dimensional lateral cephalometric radiographs.^13^ ^,^ ^14^ ^,^ ^17^ ^,^ ^19^ Shortcomings of these two-dimensional radiographs included magnification, geometric distortion, superimposition of anatomical structures and inconsistent head position.^20^ There were no studies, to the best of our knowledge, that used cone beam computed tomography (CBCT) to compare maxillary first molar distalization with Carriere distalizer appliance before and after second molar eruption.

Using CBCT to measure various skeletal and dental changes in the present study could offer the distinct advantage of one-to-one geometry, and provide the potential for utilizing additional anatomical landmarks not detectable in the two-dimensional cephalograms.^21^ ^,^ ^22^ Moreover, distinct views could be obtained for both right and left sides, allowing to increase the efficiency of image utilization, by omitting the superimposition of structures that were unrelated to the required landmark determination, and three-dimensional measurements.^22^ The multi-planer reconstruction displays of CBCT views can offer more accurate determination of cephalometric landmarks than conventional lateral cephalograms.^23^

Accordingly, the aim of this study was to compare the outcomes of maxillary first molar distalization using the Carriere distalizer appliance before and after second molar eruption. The null hypothesis was that the results of maxillary first molar distalization - including three-dimensional maxillary first molar movements, anchorage loss, amount of Class II correction and treatment duration - were not affected whether maxillary second molar was erupted or not.

## MATERIAL AND METHODS

This prospective study included two groups of patients indicated for maxillary first molar distalization (thirty patients for each group). In the first group (19 females and 11 males, mean age of 11.6± 0.9 years), the treatment was accomplished prior to the eruption of the maxillary second molar, with the follicles of the second molars placed directly toward the cervical third of the first molar root. In the second group (21 females and 9 males, mean age of 14.3 ± 1.4 years), distalization started when both maxillary first and second molars erupted.

Patients in both groups fulfilled the following inclusion criteria:

1. More than half-cusp bilateral Angle’s Class II molar relation.
2. Skeletal Class I malocclusion, with ANB angle less than 4° and YEN angle between 117° and 123°.^24, 25^
3. Total mandibular arch discrepancy, indicating that there was no need for extraction in the mandibular arch.
4. No pretreatment transverse discrepancy.
5. No previous orthodontic treatment.

The sample size was calculated according to the following formula:

$$n=\frac{2{\left(Z_{\alpha }+Z_{\left[1-\beta \right]}\right)}^{2}\times \left(\frac{SD_{1}{}^{2}+SD_{2}{}^{2}}{2}\right)}{D^{2}}$$

In which Z_α_ = 1.96 for α of 0.05 (significance at *p* ˂ 0.05) and Z_[1 - β]_ = 1.28 for β of 0.10 (the power of study is 90%). Also, SD_1_ and SD_2_ are the standard deviations of maxillary molar distalization for a pilot study of ten randomly selected patients in the first and the second groups, respectively. D is the effect size (the minimal clinical relevant maxillary molar distalization difference between both groups in the pilot study).

So, $n=\frac{2{\left(1.96+1.28\right)}^{2}\times \left(\frac{0.97^{2}+1.19^{2}}{2}\right)}{{\left(0.91\right)}^{2}}$

n= 30 patients per group.

**Figure 1:**
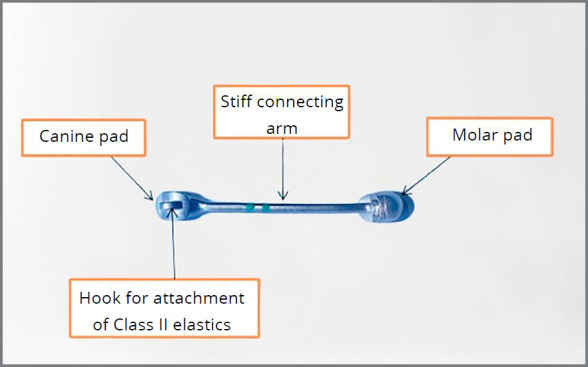
Design of the Carriere distalizer.

**Figure 2:**
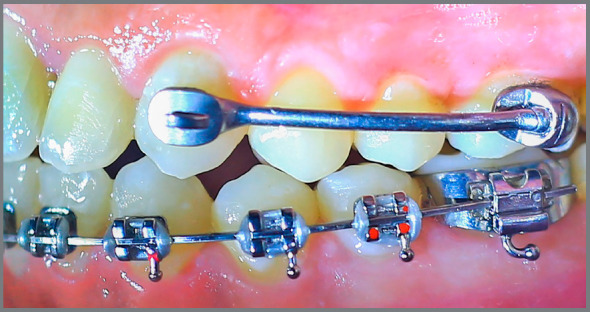
Bonded Carriere distalizer appliance.

Distalization was performed with the Carriere distalizer appliance for all patients (Figs 1 and 2). A 0.036-in lower lingual holding arch was soldered to bands cemented on the mandibular first molars, to provide anchorage for molar distalization. The mandibular arch was bonded for all patients by the same operator, using mini master brackets with 0.022-in slot size (American Orthodontics, Sheboygan, Wis) and leveled reaching 0.019 × 0.025-in stainless steel archwire. The distalizer was then bilaterally bonded by the same operator in all subjects.

All patients were instructed to use heavy Class II elastics with 1/4-in diameter (American Orthodontics, Sheboygan, Wis), attaching them from the mandibular molar band hook to the hook on maxillary cuspid pad of the distalizer. A force gauge (Dentaurum, Pforzheim, Germany) was used to measure the amount of force produced by Class II elastics once attached. Patients were instructed to wear the elastics all the time, except during eating or playing sports, and to change them after every meal. 

Every patient was instructed to fill-in a schedule, for self-reporting the duration of wearing Class II elastics every day. Follow-up visits were scheduled every two weeks, to report any problems and to check the compliance of the patients. Reports from parents were required to overcome the social desirability bias during filling-in the schedule. Patients were also instructed to fill in the report every hour, helped by memory aids to overcome the recall bias.^26^

One CBCT image (Scanora3D, Sorredex- Finland) was taken for each patient before distalization, and another one when a bilateral Class I molar relationship was attained, in the same standardized technique. Exposure was performed at 15 mA and 85 KV. The obtained CBCT images were transformed to DICOM format (Digital Imaging and Communications in Medicine) with the i-CAT software (Hatfield, Pennsylvania, USA). A fully reconstructed three-dimensional volumetric image was generated by utilizing the Mimics image processing software (Materialise Group, Leuven, Belgium).

**Figure 3:**
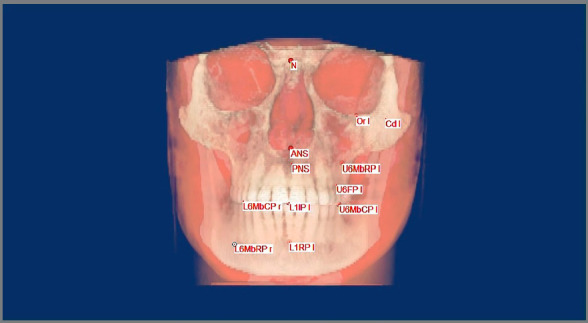
Determination of landmarks on the Mimics software.

The three-dimensional images were subsequently reoriented to the Frankfort horizontal reference plane. The sagittal reference plane was set perpendicular to the horizontal reference plane, and connecting the nasion and the right porion points. The frontal plane was extended from the nasion, and normal to the horizontal and sagittal planes. Identification of landmarks was determined by using the generated multiplanar projections. The selected points were then assessed in the three-dimensional image. Measurements were taken to compare both groups regarding the amounts of maxillary first molar distalization, mesiodistal tipping, bucccolingual torquing and rotation, in addition to anchorage loss and skeletal changes. Moreover, the treatment durations were compared. Figure 3 and Tables 1, 2 and 3 show the landmarks, planes and measurements used in this study.

**Table 1:** Three-dimensional cephalometric reference landmarks.

Point	Description
S (Sella)	The midpoint of the sella turcica
N (Nasion)	The most anterior point on the frontonasal suture
A (Subspinale)	The deepest midline point in the curved bony outline from the base to the alveolar process of the maxilla
B (Supramentale)	The most posterior point in the outer contour of the mandibular alveolar process in the median plane
M point	The center of the largest best fit circle tangent to anterior, superior, and palatal surface of premaxilla (midpoint of the premaxilla)
G point	The center of the largest best fit circle tangent to the internal, anterior, inferior, and posterior surfaces of mandibular symphysis (center of mandibular symphysis)
GnR (right gnathion)	The point in the midway between the most anterior and the most inferior points of the chin on the right side
OrR - OrL (right and left orbitale)	The most inferior point on the orbital margin at both sides
PoR (right porion)	The highest point on the external auditory meatus on the right side
ANS (anterior nasal spine)	The most anterior midpoint of the anterior nasal spine of the maxilla
PNS (posterior nasal spine)	The most posterior midpoint of the posterior nasal spine of the palatine bone
CdR-CdL (right and left Condylion)	The most superior point on the head of the condyle at both sides
U6MbCPR - U6MbCPL (right and left maxillary first molar mesiobuccal cusp tip)	The tip of the mesiobuccal cusp of the right and left maxillary first molar crowns
U6MbRPR - U6MbRPL (right and left maxillary first molar mesiobuccal root apex)	The apex of the mesiobuccal root of the right and left maxillary first molars
U6DbCPR - U6DbCPL (right and left maxillary first molar disto-buccal cusp tip)	The tip of the distobuccal cusp of the right and left maxillary first molar crowns
L6MbCPR - L6MbCPL (right and left mandibular first molar mesiobuccal cusp tip)	The tip of the mesiobuccal cusp of the right and left mandibular first molar crowns
U6FPR - U6FPL (right and left maxillary first molar furcation point)	The mid furcation point between the roots of the right and left maxillary first molars
L6MbRPR - L6MbRPL (right and left mandibular first molar mesiobuccal root apex)	The apex of the mesiobuccal root of the right and left mandibular first molars
L1IPR - L1IPL (right and left mandibular central incisor incisal point)	The tip of the incisal edge of each mandibular central incisor
L1RPR - L1RPL (right and left mandibular central incisor root point)	The apex of the root of each mandibular central incisor

**Table 2:** Three-dimensional cephalometric reference lines and planes.

Line or plane	Description
FHP (Frankfurt horizontal plane)	The plane passing through OrR, OrL and PoR points
VP (Vertical plane)	The plane passing through CdR and CdL and perpendicular to the FHP
MxS (Maxillary sagittal line)	The line connecting ANS and PNS
FL (Frontal line)	The line connecting OrR and OrL
U6 long axis	The line connecting U6MbCP and U6MbRP
L6 long axis	The line connecting L6MbCP and L6MbRP
L1 long axis	The line connecting L1IP and L1RP

**Table 3:** Three-dimensional CBCT measurements.

Measurement	Description
SNA	The angle between SN and NA lines
CdR - ANS	The distance between the right Condylion and the anterior nasal spine
Anteroposterior position of the maxilla	SNApre - SNApost (CdR - ANS)pre - (CdR - ANS)post
SNB	The angle between SN and NB lines
CdR - GnR	The distance between the right Condylion and the right Gnathion
Anteroposterior position of the mandible	SNBpost - SNBpre (CdR - GnR)post - (CdR - GnR)pre
ANB	The difference between SNB and SNA angles
YEN angle	angle formed between SM line and MG line
Anteroposterior relationship between maxilla and mandible	ANBpre - ANBpost YEN anglepre - YEN anglepost
U6 AP (maxillary first molar antero-posterior position)	Measured as the perpendicular distance from (U6MbCPR or U6MbCPL) to the VP (Vertical plane)
Maxillary first molar distalization	U6 APPre - U6 APPost
U6 VP (maxillary first molar vertical position)	Measured as the perpendicular distance from (U6FPR or U6FPL) to the FHP (Frankfurt horizontal plane)
Maxillary first molar intrusion	U6 VPPre - U6 VPPost
U6 MD (maxillary first molar mesio-distal angulation)	Measured as the posterior angle between the U6 long axis and the MxS (Maxillary sagittal line)
U6 mesio-distal angulation change	U6 MDPre - U6 MDPost
U6 BL (maxillary first molar bucco-lingual inclination)	Measured as the external downward angle between the U6 long axis and the FL (Frontal line)
U6 bucco-lingual inclination change	U6 BLPost - U6 BLPre
U6 ROT (maxillary first molar rotation)	Measured as the internal angle between the line connecting the U6MbCP and U6DbCP and the MxS (Maxillary sagittal line)
Maxillary molar rotation	U6 ROTPre - U6 ROTPost
L6 AP (mandibular first molar antero-posterior position)	Measured as the perpendicular distance from (L6MbCPR or L6MbCPL) to the VP (Vertical plane)
Mandibular molar mesialization	L6 APPost - L6 APPre
L6 MD (mandibular first molar mesio-distal angulation)	Measured as the posterior angle between the L6 long axis and the MxS (Maxillary sagittal line)
Mandibular molar mesio-distal angulation change	L6 MDPre - L6 MDPost
L1 BL (mandibular central incisor bucco-lingual inclination)	Measured as the anterior angle between the L1 long axis and the MxS (Maxillary sagittal line)
Mandibular incisor bucco-lingual inclination change	L1 BLPost - L1 BLPre

Before starting distalization in both groups, the severity of the Class II molar relationship was measured as the horizontal distance between the mesiobuccal cusp tips of maxillary and mandibular first molars, and compared in both groups. The distance zero indicated a half-cusp Class II molar relationship. As the distance increased, the severity of Class II relationship increased. Complementarily, the skeletal relationship was compared between both groups. Independent *t*-test revealed no significant differences between both groups in all these pretreatment variables (pretreatment Class II severity was 2.5 ± 0.8 mm and 2.2 ± 0.9 mm, respectively, with *p*-value = 0.201; ANB angle was 2.8 ± 0.5° and 2.9 ± 0.8°, respectively, with *p*-value = 0.471; and YEN angle was 120.1 ± 2° and 119.7 ± 1.7°, respectively, with *p*-value = 0.352).

In all patients of both groups, bilateral Class I molar relationship was achieved. All patients in both groups properly tolerated the appliance. No distalizer debonding occurred in any subject from any group during the treatment period.

## STATISTICAL METHOD

The collected data were statistically analyzed using SPSS (Statistical Package for Social Sciences) software (version 9.0, SPSS, Chicago, USA). Descriptive statistics were done (means and standard deviations) for all variables included in the study.

All variables were subjected to Shapiro-Wilk test, which revealed normal distribution for all of them (*p*˃ 0.05 for all variables). Analyses between both groups for parametric quantitative data were done using independent samples *t*-test, and for qualitative data, using Chi-square test (expected number per cell > 5). The level of significance was defined at *p* value < 0.05.

Correlations between pretreatment Class II severity and other variables were analyzed using Pearson’s correlation coefficient. Differences with less than 5% probabilities were considered statistically significant.

## ERROR OF THE METHOD

All reference landmarks, planes and measurements were relocated and measured again by three different operators. Reliability of measurements was estimated by Cronbach’s Alpha and Inter-Class Correlation.

## RESULTS

The method reliability was excellent, with Cronbach’s Alpha and Inter-Class Correlation of more than 0.9 for all measurements in both groups (Table 4). For all variables included in the study, no significant differences were found between boys and girls in both groups (Table 5). Accordingly, for both groups, the results for both boys and girls were analyzed together.

**Table 4:** Estimation of the reliability of measurements in both groups, by Cronbach’s Alpha and Inter-Class Correlation.

	Group II			Group I		
	Cronbach’s Alpha	Interclass correlation		Cronbach’s Alpha	Interclass correlation	
		R	P value		R	P value
U 6 AP	0.999	0.998	<0.001*	0.999	0.996	<0.001*
Pre-treatment Class II severity	0.999	0.998	<0.001*	0.998	0.993	<0.001*
U 6 VP	0.998	0.993	<0.001*	0.999	0.996	<0.001*
U 6 MD	0.999	0.998	<0.001*	1	0.999	<0.001*
U 6 BL	1	0.999	<0.001*	1	0.999	<0.001*
U 6 ROT	0.998	0.993	<0.001*	0.999	0.998	<0.001*
L 6 AP	0.999	0.996	<0.001*	0.998	0.993	<0.001*
L 6 MD	1	0.999	<0.001*	0.999	0.996	<0.001*
L 1 BL	0.998	0.993	<0.001*	1	0.999	<0.001*
SNA	0.999	0.998	<0.001*	0.998	0.993	<0.001*
CdR - ANS	0.988	0.964	<0.001*	0.998	0.994	<0.001*
SNB	0.998	0.994	<0.001*	1	0.999	<0.001*
CdR - GnR	0.999	0.998	<0.001*	0.999	0998	<0.001*
ANB	0.998	0.994	<0.001*	0.998	0.993	<0.001*
YEN angle	1	0.999	<0.001*	0.999	0.998	<0.001*

*: Significant level at P value < 0.05.

**Table 5:** Difference between boys and girls, for both groups.

	Group I			Group II		
	Males	Females	P value	Males	Females	P value
Pretreatment Class II severity	2.9±0.9	2.3±0.7	0.127	2.3±1.1	2±0.5	0.558
U 6 AP	4.2±0.8	3.8±0.8	0.301	3.3±0.6	2.7±0.6	0.078
Percent of first molar movement	70.2±14.1	65.7±16.6	0.952	62.2±11.4	53.8±14.3	0.209
U 6 VP	1.3±0.9	0.9±0.8	0.874	1.6±0.8	1.3±0.8	0.634
U 6 MD	5±1.5	5.5±0.9	0.440	4.8±1.2	4.9±1.6	0.917
U 6 BL	3±1.1	2.9±0.9	0.367	2.9±0.9	3.3±0.8	0.905
U 6 ROT	5.6±1.4	5.5±0.7	0.353	6.9±1	7.4±1.2	0.800
L 6 AP	1 ±0.8	1.2±0.9	0.958	2.1±0.9	1.8±0.7	0.491
L 6 MD	3±0.7	3.4±1.7	0.233	4.3±1.2	5.2±1.3	0.536
L 1 BL	4.7±1.2	5±1.9	0.526	6.6±0.9	6.9±1.3	0.634
SNA	0.7±0.3	0.9±0.4	0.711	0.8±0.4	0.9±0.3	0.916
CdR - ANS	0.7±0.4	1±0.5	0.634	1.1±0.4	1.4±0.6	0.874
SNB	1±0.4	0.8±0.3	0.204	0.8±0.3	1±0.5	0.427
CdR - GnR	3.8±1.2	3.5±1.1	0.543	4.1±0.7	4.1±1	0.899
ANB	1.8±0.4	1.5±0.4	0.143	1.6±0.3	1.9±0.4	0.175
YEN angle	4.9±1.1	4.9±1.3	0.988	5.4±0.8	5.5±1.6	0.904
Elastics wearing time (hours/ day)	19.6±1.3	20±1.3	0.544	20.9±1.2	21±0.9	0.816
Treatment Duration (weeks)	18.7±2	19.3±1.6	0.542	23.5±1.4	22.9±1.6	0.404

Quantitative data expressed as mean ± SD.

Chi square test for qualitative data between both groups.

Independent samples *t*-test for quantitative data between both groups.

Significant level at *p*-value < 0.05.

No significant difference (*p*= 0.252) was found in the mean duration of elastics wear per day between both groups (19.8 ± 2 and 20.9 ± 1.5 hours per day, respectively). Also, no significant difference was found (*p*= 0.32) in the amount of force produced by Class II elastics between both groups (194 ± 26g and 201 ± 31 g, respectively).

The mean first molar distalization period in the first group was 19.2 ± 1.6 weeks. It was significantly smaller (*p*= 0.001) than the mean distalization period in the second group, which was 23.3 ± 2.3 weeks. No significant differences between both groups were observed in all skeletal measurements. No significant correlations between the pretreatment Class II severity and other variables included in the study were observed (Table 6).

**Table 6:** Correlation between the pretreatment Class II severity and other variables included in the study, in both groups.

	Group I		Group II	
	r	P value	r	P value
U 6 AP	0.403	0.121	0.409	0.116
U 6 VP	-0.137	0.614	0.030	0.911
U 6 MD	0.0	1	0.166	0.540
U 6 BL	-0.007	0.979	0.029	0.915
U 6 ROT	0.083	0.761	-0.031	0.910
L 6 AP	0.068	0.803	-0.301	0.257
L 6 MD	-0.087	0.748	0.240	0.370
L 1 BL	-0.242	0.367	0.171	0.527
SNA	-0.026	0.924	-0.467	0.068
CdR - ANS	-0.133	0.624	-0.117	0.667
SNB	0.081	0.765	0.140	0.604
CdR - GnR	0.345	0.191	0.247	0.356
ANB	-0.334	0.207	0.335	0.204
YEN angle	-0.124	0.648	-0.108	0.690
Elastics wearing time (hours/ day)	0.076	0.780	0.149	0.582
Treatment duration (weeks)	-0.031	0.908	0.046	0.866

Pearson’s correlation.

*: Significant level at P value < 0.05.

Maxillary first molar distalization constituted 67.4 ± 15.1% from the total Class II correction in the first group, which was significantly greater than in the second group, that was 58.5 ± 13% (*p*= 0.022).

The amount of maxillary first molar distalization was significantly greater (*p*= 0.001) in the first than the second group (3.9 ± 0.8 and 3 ± 0.6 mm, respectively). No statistically significant differences were found between both groups regarding the quantities of maxillary first molar intrusion, mesiodistal tipping and buccolingual torquing.

Regarding maxillary first molar rotation, distalizing both first and second molars together resulted in more significant first molar rotation than distalizing the first molar alone (*p*˂ 0.001).

The mandibular incisor labiolingual torquing and the mandibular first molar mesialization and mesiodistal tipping were significantly increased in the second group, indicating more anchorage loss. All these results are summarized in Table 7.

**Table 7:** Changes in three-dimensional cephalometric measurements, elastics wearing time and treatment duration after maxillary first molar distalization, in both groups.

	Group I	Group II	P value
Sex	Male 11 (36.67%) Female 19 (63.33%)	Male 9 (30%) Female 21 (70%)	0.480
Pretreatment Class II severity	2.5±0.8	2.2±0.9	0.201
U 6 AP	3.9±0.8	3±0.6	0.001*
Percent of first molar movement	67.4±15.1	58.5±13	0.022*
U 6 VP	1.2±0.8	1.5±0.7	0.323
U 6 MD	5.2±1.2	4.9±1.4	0.402
U 6 BL	3±1	3.1±0.8	0.605
U 6 ROT	5.6±1.1	7.2±1.1	<0.001*
L 6 AP	1.1±0.7	2±0.8	0.004*
L 6 MD	3.2±1.2	4.8±1.3	0.001*
L 1 BL	4.8±1.5	6.7±1.1	<0.001*
SNA	0.8±0.5	0.8±0.4	0.692
CdR - ANS	0.9±0.4	1.3±0.5	0.250
SNB	0.9±0.4	0.9±0.5	0.763
CdR - GnR	3.6±1.1	4.1±0.8	0.195
ANB	1.7±0.4	1.8±0.4	0.519
YEN angle	4.9±1.2	5.4±1.2	0.212
Elastics wearing time (hours/ day)	19.8±2	20.9±1.5	0.252
Treatment Duration (weeks)	19.2±1.6	23.3±2.3	0.001*

Quantitative data expressed as mean ± SD while qualitative data expressed by frequency and percentage.

Chi-square test for qualitative data between both groups.

Independent samples *t-*test for quantitative data between the two groups.

* Significant level at *p-*value < 0.05.

## DISCUSSION

Attaining a Class I molar relationship is a fundamental component of appropriate balanced occlusion and facial esthetics.^27^ There are controversies regarding the influence of second molar eruption upon various aspects of maxillary first molar distalization. The results of this study did not show any statistically significant differences in different skeletal measurements whether second molar had erupted or not.

The treatment time was significantly shorter, the quantity of maxillary first molar distalization was significantly larger and the amount of rotation was significantly smaller in the group with unerupted maxillary second molar. The differences between both groups were not significant regarding the amount of first molar buccolingual torquing.

Also, the amount of anchorage loss (indicated by mandibular first molar mesial migration and mandibular incisor labiolingual inclination) was significantly greater when the second molar was erupted.

The main outcomes of this study corroborate the results of two lateral cephalometric studies utilizing intra-arch NiTi coil spring with Nance appliance^15^ and molar distalizing bow.^28^ This approach is efficient to distalize maxillary first molar prior to second molar eruption, attaining the advantages of more efficient first molar distalization and less anchorage loss. Continuing maxillary first molar distalization following maxillary second molar eruption slows down the rate of distalization, that becomes equivalent to starting first molar distalization after second molar eruption.^17^

However, according to two other studies using the XBow appliance^13^ and the Pendulum appliance,^29^ lateral cephalometric measurements did not show significant differences in the quantity of maxillary first molar distalization and anchorage loss whether the second molar was erupted or not, suggesting that second molar eruption has negligible influence on first molar distalization. 

Also, the results of this study support the concept that it is more hazardous to the anchorage if both first and second molars are distalized together, as combined teeth have larger root surface area than a single tooth. Anchorage is less compromised when the first molar is distalized before second molar eruption, resulting in less time-consuming correction of the anchorage loss.^15^

However, mesiodistal tipping of the first molar was not significantly changed in this study, whether distalized before or after second molar eruption. These findings agree with the results of a lateral cephalometric study using the XBow appliance, which concluded that there was no difference in the mesiodistal tipping change of the distalized maxillary first molar whether the second molar was present or unerupted.^13^

This evidence does not agree with the idea that the unerupted second molar would probably impact distal tipping of the first molar to a considerable degree,^14^ resulting in more significant first molar mesiodistal tipping than when the second molar is present.^5^ Both studies utilized two-dimensional lateral cephalograms to assess alterations in the position of the maxillary first molar.^5^ ^,^ ^14^

Concerning maxillary first molar buccolingual torquing, this study suggests no significant difference whether the first molar is distalized before or after second molar eruption. According a three-dimensional finite element analysis by Kang et al.,^30^ it was more effective to utilize a bone-anchored pendulum appliance to distalize maxillary first molar before second molar eruption, as this resulted in less first molar buccal tipping.

According to the results of this study, if the operator has the choice to distalize maxillary first molars with the Carriere distalizer appliance before or after second molar eruption, earlier initiation of the treatment is more favorable. 

As maxillary first molars in all subjects included in this study were distalized with Carriere distalizer appliance, outcomes of this study can be considered precise for patients treated with this distalizer only.

In this study, randomization of patients between both groups implicates that all subjects should have unerupted second molars, starting treatment immediately in the first group and waiting for second molar eruption in the second group. As delaying the treatment was not ethical for the second group, subjects were selected in both groups by a single operator depending on the predetermined selection criteria, except that second molars had already erupted in one group.

## CONCLUSION

Maxillary first molar distalization using Carriere distalizer appliance before maxillary second molar eruption is more efficient, less time-consuming and more anchorage-conserving than after second molar eruption.
